# Evolution of *piggyBac* Transposons in Apoidea

**DOI:** 10.3390/insects14040402

**Published:** 2023-04-21

**Authors:** Xueyuan Li, Zhongxia Guan, Feng Wang, Yali Wang, Emmanuel Asare, Shasha Shi, Zheguang Lin, Ting Ji, Bo Gao, Chengyi Song

**Affiliations:** College of Animal Science and Technology, Yangzhou University, Yangzhou 225009, China

**Keywords:** Apoidea, *piggyBac*, transposons, evolution

## Abstract

**Simple Summary:**

Bees are one of the mega-diverse class of insects in Hymenoptera. As predators and the main crops pollinator, bees play an essential role in improving crop yield and the quality whiles providing substantial economic importance. Transposable elements (TEs) exist widely in nature and they make up a significant portion of prokaryotic and eukaryotic genomes. *piggyBac* (*PB*) transposons, belonging to DNA transposons, have been identified in plants, fungi, and animals. The advantages of the modified *PB* transposon system or high transposable efficiency and broad host ranges have led to their widespread application in transgenesis, mutagenesis, and gene therapy. At present, there is no systematic report on *PB* transposon in bees. Therefore, this study focused on annotating the transposons of the *PB* superfamily in bees to reveal the distribution, diversity, structural characteristics, evolutionary pattern and activity of *PB* transposons in the available genomes of bees.

**Abstract:**

In this study, we investigated the presence of *piggyBac* (*PB*) transposons in 44 bee genomes from the Apoidea order, which is a superfamily within the Hymenoptera, which includes a large number of bee species crucial for pollination. We annotated the *PB* transposons in these 44 bee genomes and examined their evolution profiles, including structural characteristics, distribution, diversity, activity, and abundance. The mined *PB* transposons were divided into three clades, with uneven distribution in each genus of *PB* transposons in Apoidea. The complete *PB* transposons we discovered are around 2.23–3.52 kb in length and encode transposases of approximately 580 aa, with terminal inverted repeats (TIRs) of about 14 bp and 4 bp (TTAA) target-site duplications. Long TIRs (200 bp, 201 bp, and 493 bp) were also detected in some species of bees. The DDD domains of the three transposon types were more conserved, while the other protein domains were less conserved. Generally, most *PB* transposons showed low abundance in the genomes of Apoidea. Divergent evolution dynamics of *PB* were observed in the genomes of Apoidea. *PB* transposons in some identified species were relatively young, whiles others were older and with some either active or inactive. In addition, multiple invasions of *PB* were also detected in some genomes of Apoidea. Our findings highlight the contribution of *PB* transposons to genomic variation in these species and suggest their potential as candidates for future gene transfer tools.

## 1. Introduction

The Apoidea superfamily, which originated from the early-to-middle Cretaceous period, is one of the main categories of Hymenoptera Apocrita [1]. Apoidea has various morphological characteristics as a large group of holometabolous insects [2,3,4]. Bees are known for their short generation cycle, strong reproduction, parthenogenesis, and captive-bred. These insects have been extensively studied and can be used as model animals for studying the evolution of sociality, intra- and interspecific communication, and physiological adaptations [2,5,6,7,8,9,10]. Bees exhibit three different ways of life: sociality, solitary, and parasitism, with the majority being solitary [11,12]. Most bees are important pollinators of angiosperms and play crucial roles in ecosystem functions [3,4]. Apoidea insects directly impact 6% of the 128 most important crops consumed worldwide [13]. The Apoidea superfamily comprises seven widely recognized families: Megachilidae, Apidae, Andrenidae, Colletidae, Halictidae, Melittidae, and Stenotritidae [1,12,14].

Transposable elements (TEs) are DNA sequences that can be independently copied or broken from their original site and inserted into another site, playing a regulatory role in the evolution and gene regulation of all organisms. They ‘jump’ from one position in the genome to another through a series of processes, such as cutting and reintegrating, also called the jumping gene. The TE generally moves the TE sequence itself and is not dependent on the sequence relationship between the donor and recipient. TEs have been detected in various organisms, including bacteria, fungi, and insects [15], and they account for 15–47% of insect genomes, 35–69% of mammalian genomes, and about 90% of some plant genomes [16,17,18,19,20,21,22,23,24,25]. TEs also play important roles in shaping genome evolution and transcriptome regulation [15,26,27,28,29].

Tes are divided into two categories based on their transposable mechanisms. Class I or retrotransposons rely on RNA intermediates and transpose via a ‘copy-and-paste’ mechanism, whereas Class II or DNA transposons use DNA intermediates and usually transpose by ‘cut-and-paste’ mechanisms [30,31,32]. Several diverse DNA transposon superfamilies, such as *piggyBac*, *Tc1/mariner*, *pogo*, *hAT*, *Helitron*, and *PIF-Harbinger*, have been reported, and their evolutionary profiles have been well-defined for some groups, such as *Tc1/mariner* and *pogo* DNA transposons [12].

The active *piggyBac* (*PB*) transposon was initially discovered in the Lepidopteran cabbage looper moth (*Trichoplusia ni*), and subsequent genome sequencing and comparison across multiple species revealed its high diversity and widespread distribution [33,34]. *PB* elements have been identified in various eukaryote kingdoms, including fungi, plants, and animals (insects, crustaceans, amphibians, fishes, and mammals) [35,36]. The active *PB* transposon from *T. ni* has been developed into an efficient gene transfer vector [35] with potential applications in human gene therapy [37,38,39,40]. The *PB* transposon from *T. ni* is 2472 bp in length and contains TTAA target sites, with 14 bp terminal inverted repeats (TIR) and 19 bp sub-terminal asymmetric inverted repeats (STIR) located at 3 and 31 bp from the 5′ and 3′ TIRs, respectively [33,36,41,42]. It has an open reading frame (ORF) in the middle, which is 1782 bp long, encoding a protein of 594 amino acids with a molecular weight of 64 kDa [36,43]. *PB* transposases belong to DD [E/D]-transposases [44] and are currently defined using five domains, namely the N-terminal domain (NTD), the Dimerization and DNA-binding domain 1 (DDBD1), the catalytic domain (also named DDE/DDD) and the DDBD2 and C-terminal cysteine-rich domain (CRD) [45]. In addition, there is little obvious sequence similarity between the *PB* superfamily and other transposon superfamilies [35,44]. The majority of mammalian genomes, such as the human, mouse, rat, and dog genomes, contain decayed *piggyBac* transposons [21,36,46,47,48]. However, two families of *piggyBac*-like elements were found in *Myotis lucifugus*, and display recent activity, forming a strong cluster with the sea squirt, *Ciona intestinalis* [49]. Another study identified *PB* elements in *Microcebus murinus*, divided them into three categories, and found that those three types of *PB* transposons were relatively young [50]. In addition, 32 *piggyBac*-like elements in the red flour beetle *Tribolium castaneumre* are divided into 14 diverse groups [51]. Moreover, at least five domesticated *piggyBac* (PGBD) have been found in the human genome [36]. These findings highlight the wide distribution and high diversity of *PB* transposons, although their internal classification at the family level remains largely unknown.

Currently, there is limited information on DNA transposons in bees, with only *pogo* and *mariner*, belonging to the *ITm* group (*IS630-Tc1-Mariner*), being well-defined in bees [12,52]. In this paper, we investigated the genomes of 44 species of Apoidea and annotated the *PB* transposons in each species to determine their structural characteristics, overall distribution, and phylogenetic location and classification, and conducted evolutionary dynamics analysis. Our data shed light on the evolutionary pattern of *PB* transposons in Apoidea and enhanced our understanding of their contribution to the evolution of the Apoidea genome.

## 2. Materials and Methods

### 2.1. piggyBac Mining in Apoidea

All reference *PB* transposase sequences [36] were used to search the genomes of Apoidea in the WGS (whole-genome shotgun contigs) database of NCBI (https://www.ncbi.nlm.nih.gov, accessed on 22 November 2022) using TBLASTN (v. 2.12.0) [53] with the value of 1e-100. The best hits of TBLASTN [53] were extended to 2000 bp flanks, which were then BLASTN against the genome. The hits were downloaded and aligned to determine the boundaries of these transposons in each genome. The boundaries of the transposons (TIRs and TSDs) were manually checked, combined with the help of FastPCR (https://primerdigital.com/fastpcr.html, accessed on 25 November 2022) [54] and the Bioedit program (v.7.2.0) [55]. The representative sequences or consensus sequences (derived for *PB* > 10 copies in genome) of transposons in each genome were used to determine the genomic copies (coverage > 40% and similarity > 80% of queries) by BLAST.

### 2.2. Sequence Analysis and Phylogenetic Analysis

The transposases of the mined transposons were predicted by Genescan (https://www.bing.com/search?q=genescan, accessed on 29 November 2022), and the sequence identities were calculated using the BioEdit tool [55]. The sequence identity matrix was drawn with the HeatMap in the TBtools (v. 1.0987663) (https://github.com/CJ-Chen/TBtools/releases, accessed on 30 January 2023) [56]. The *PB* transposases were aligned using MAFFT [57] and manually edited by Bioedit [52]. The structure predicted by the transposase was presented by the tool Illustrator for Biological Sequences (IBS v. 1.0.3) [58]. The online website Welogo v.3.7.12 (http://weblogo.threeplusone.com/create.cgi, accessed on 16 December 2022) was used to create logo images of TIRs.

Overall, 13 *PB* transposase reference sequences collected from the previous report [36] and 10 IS1380 transposases downloaded from ISfinder (https://www-is.biotoul.fr/scripts/search-db.php, accessed on 23 January 2023) [59], which are distantly related transposases from prokaryotes [60], combined with the mined *PB* transposases in Apoidea, together submitted for phylogenetic analysis. A multiple sequence alignment was carried out by using the G-INS-I method of MAFFT (v7.310) [57]; then, the maximum likelihood method was used to construct the phylogenetic tree using IQ-TREE (v. 1.6.1), with an ultrafast bootstrap value of 1000. According to the Bayesian information criterion, the most suitable amino acid substitution model for these data was the VT+F+G4 model that was optimally matched according to BIC selected by ModelFinder embedded with the IQ-TREE program [61,62].

### 2.3. Evolutionary Dynamics Analysis

The *PB* transposon in each genome was annotated using the RepeatMasker program (http://www.repeatmasker.org/RMDownload.html, accessed on 20 December 2022). Then, the Kimura (K) divergence was calculated using the calcDivergenceFromAlign.pl package from RepeatMasker [63]. The Kimura (K) divergence reflects the activity of transposons on the relative time scale of each genome [64]. It is generally believed that young transposons have lower Kimura (K) divergence [65].

## 3. Results

### 3.1. Distribution of piggyBac Transposons in Apoidea

**Figure 1:**
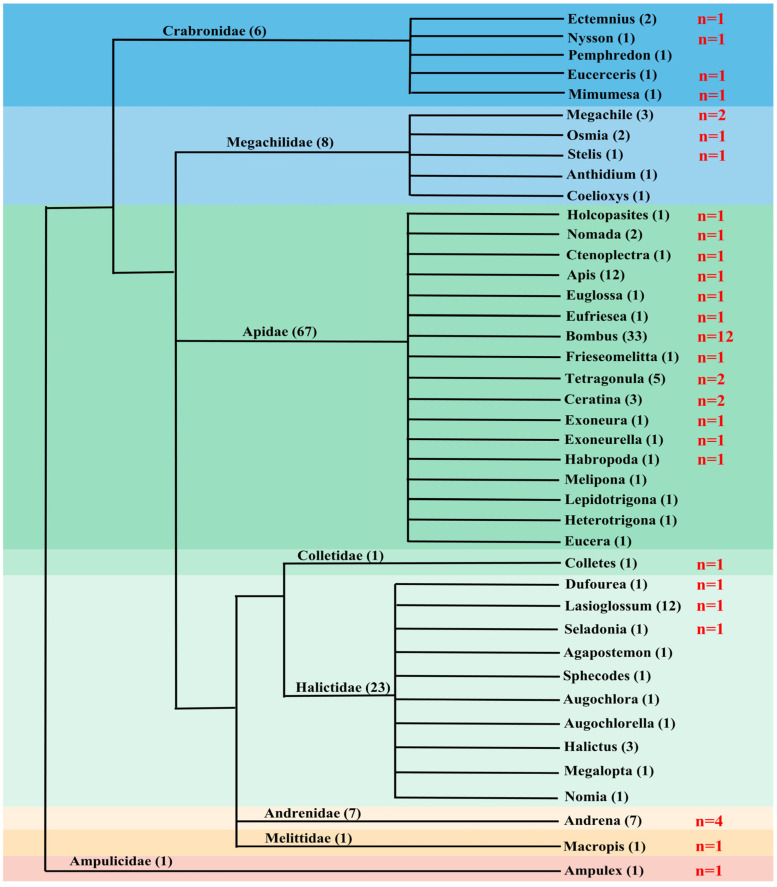
Distribution of *piggyBac* (*PB*) transposons in Apoidea among different genera. The tree on the left is the phylogenetic relationship between all genera in which transposons were detected, and different colors represent different families. The numbers in parentheses are the number of species in the families and genera used to examine, and “*n*” represents the number of species with *PB* transposons detected in each genus.

*PB* transposons showed an uneven distribution across the genus of Apoidea, as summarized in Figure 1 and Supplementary Table S1. Out of the 127 species examined, we detected a total of 44 *PB* elements in 44 species (34.65%) belonging to 27 genera within eight families of Apoidea. The majority of *PB* transposons were found in Apidae, with 26 species (38.81%) of Apidae containing *PB* transposons, while other families had fewer detections, such as three species (13.04%) in Halictidae, four species (66.67%) in Crabronidae, four species (57.14%) in Andrenidae, and four species (50%) in Megachilidae. *PB* transposons were only detected in one species in each of the following three families of bees, Colletidae, Melittidae, and Ampulicidae (Figure 1 and Supplementary Table S1).

Among the 27 genera, *PB* transposons were found in 12 species (36.36%) of *Bombus*, four species (57.14%) of *Andrena*, two species (66.67%) of *Ceratina*, two species (66.67%) of *Megachile*, and two species (40%) of *Tetragonula*. In some lineages, *PB* transposons were detected in only one species (Figure 1 and Supplementary Table S1).

### 3.2. Low Abundance of piggyBac Transposons in Apoidea

The copy number of *PB* in each genome was investigated by BLAST as described in the methods. Our data revealed significant variation in the copy numbers of *PB* transposons among different species, ranging from 1 (e.g., *Ceratina calcata*, *Seladonia tumulorum*) to 335 (*Eufriesea mexicana*) in the 44 species harboring *PB* elements (Table 1 and Supplementary Table S1). Generally, *PB* transposons were found in low copy numbers in most host genomes of Apoidea, with 28 host genomes containing less than 10 copies of *PB*. Only four species have more than 50 copies of *PB* in their genome (e.g., *Bombus soroeensis*, *Bombus terrestris*, *Andrena haemorrhoa*, and *E. mexicana*). Among these, very high copy numbers were identified in the genomes of *A. haemorrhoa* (236) and *E. mexicana* (335). Additionally, most *PB* transposons existed as truncated copies, with only 20 species harboring intact *PB* transposons (encoding transposase longer than 500 amino acids and detectable two-end TIRs), which accounted for 45.45% of the species containing *PB* copies. Although some species had high full copy numbers of *PB*, the intact copies were very low or not detectable. For example, *B. terrestris* contained 86 copies of *PB*, but only two of them were intact copies, while *A. haemorrhoa*, *B. soroeensis*, and *E. mexicana* had totals of 236, 58 and 335 in copy numbers, respectively, but intact copies were not detected in these genomes. In general, *PB* transposons were not significantly amplified in bees, and most species displayed a low abundance of *PB* (Table 1 and Supplementary Table S1).

### 3.3. Structural Organization of piggyBac Transposons in Apoidea

**Figure 2:**
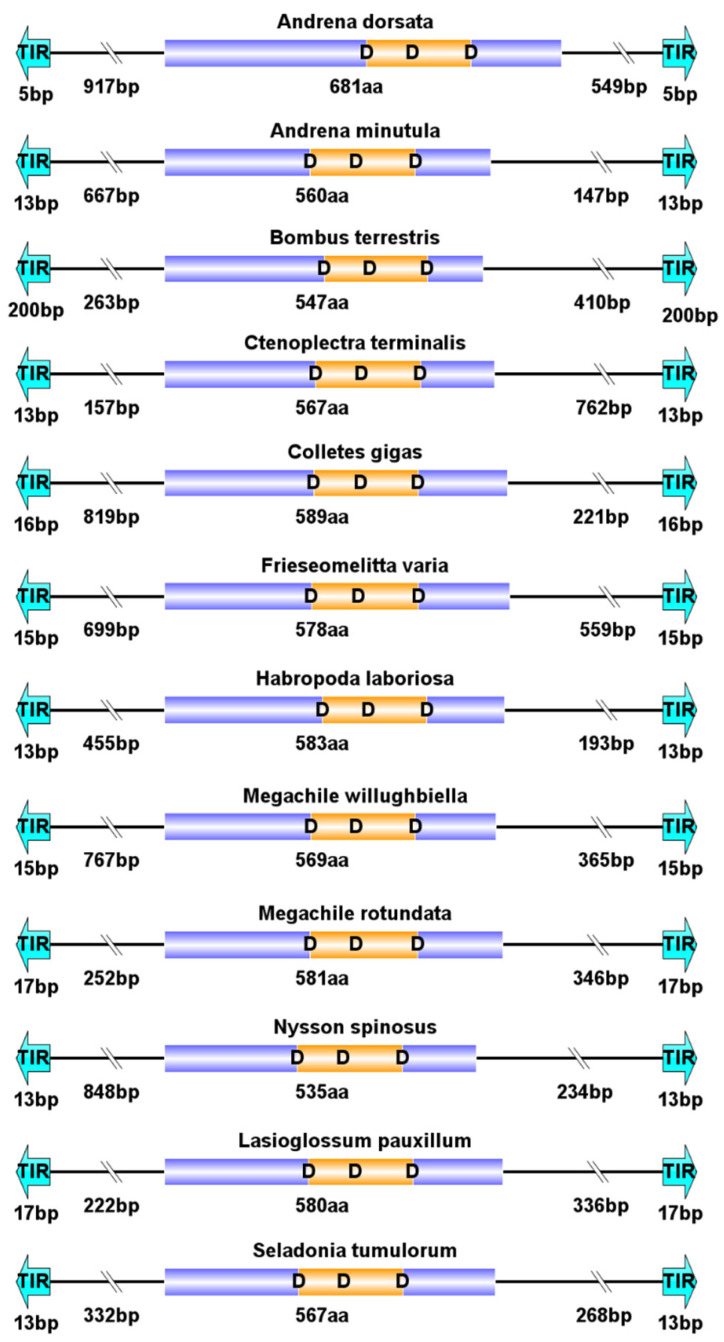
Structural organization of complete and representative *piggyBac* (*PB*) elements in Apoidea. (1) The bright blue part represents the terminal inverted repeats (TIRs) at both ends of the *PB* transposons; (2) The purple part represents the ORF; (3) The yellow part in the middle represents the catalytic domains; (4) The corresponding length of each section is marked below.

**Figure 3:**
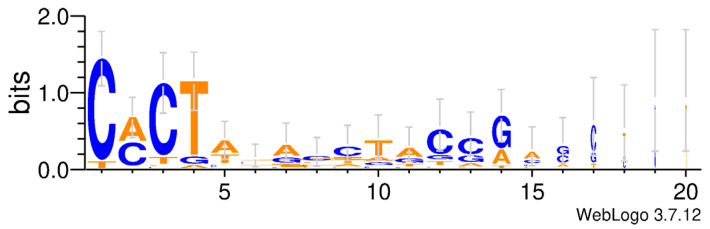
The logo figure of *piggyBac* (*PB*) Transposons’ TIR (*n* = 40). The online website Welogo v.3.7.12 (http://weblogo.threeplusone.com/create.cgi, accessed on 16 December 2022) was used to create logo images of the TIR sequences. The value 2 on the y axis stands for maximum possible frequency.

The total length of *PB* transposons ranges from 1.37 kb to 3.52 kb, and they contain a single ORF encoding for the transposase, flanked by TIRs and TSDs (Figure 2). Some species, such as *Bombus bifarius*, *Ceratina calcarata*, *Frieseomelitta varia*, and *Andrena dorsata*, have longer *PB* transposons (>3 kb) (Table 2 and Supplementary Table S1). Among them, *A. dorsata* has the longest transposon length (3518 bp) and encodes the longest transposase (681 aa). Most *PB* transposons carry short TIRs (<25 bp); however, few *PB* transposons harbor long TIRs, such as *PB* in *B. bifarius* (TIR: 201 bp), *B. terrestris* (TIR: 200 bp) and *Exoneura robusta* (TIR: 489 bp). The conserved and consistent TSD (TTAA) was observed in most *PB* transposons (Table 2 and Supplementary Table S1). The sequence logo of TIRs revealed two conserved motifs (CACTA and TACCG) in the TIRs, with the CACTA motif being the most highly conserved at the TIR end across these *PB* elements (Figure 3). High similarity between the left and right TIRs of each element was observed, displaying 100% sequence identity in 21 species (Supplementary Table S1).

### 3.4. Transposase Domain Organization

**Figure 4:**
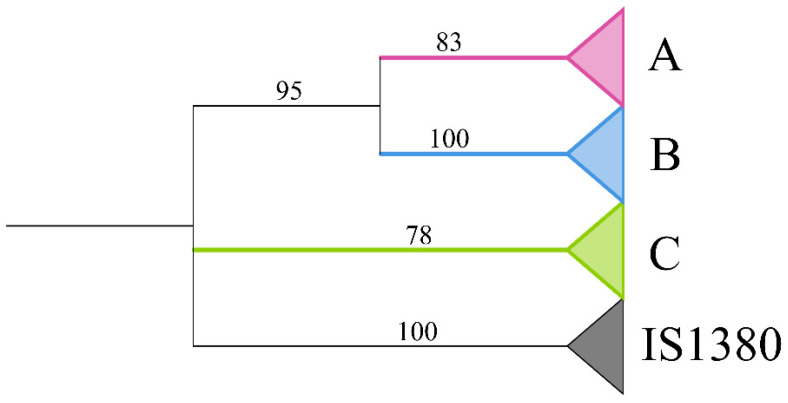
Phylogenetic tree of *piggyBac* (*PB*) elements in Apoidea identified in this study together with 24 reference sequences. The sequences of IS1380 were used as the outgroup. Red, blue, and green, respectively, represented three types of *PB* transposons (A–C). The complete evolutionary tree is in Figure S1.

The evolutionary tree of the excavated *PB* transposases (≥100 aa) was constructed using the IQ-tree program [61], with 24 reference sequences [36], and the IS1380 transposases set as an outgroup. The phylogenetic tree revealed three distinct clades (A, B, and C) of *PB* transposases in Apoidea with robust bootstrap supports (≥78%), as summarized in Figure 4 and Supplementary Figure S1.

**Figure 5:**
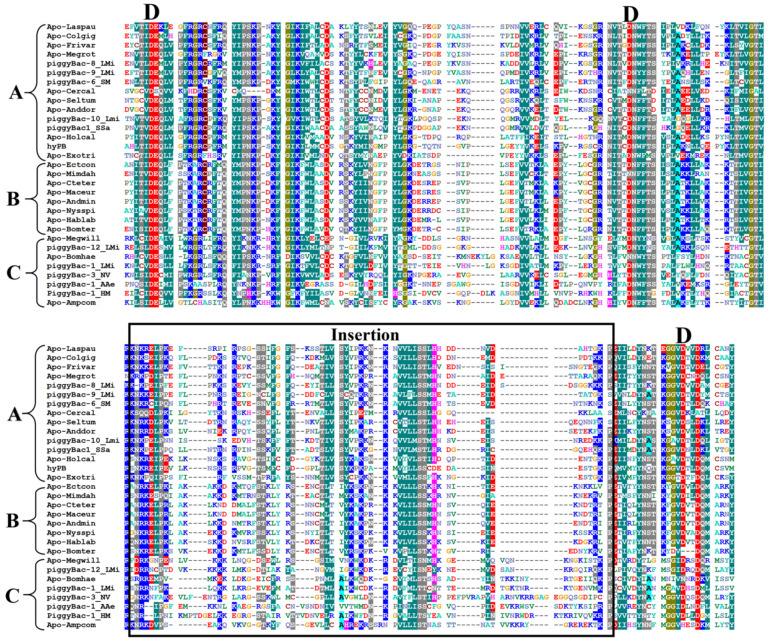
Multiple sequence alignments of the DDD domains of complete *piggyBac* (*PB*) transposases (≥ 500 aa) of three classes. The black frame represents the insertion motif of the DDD domain, and different conserved residues are colored differently. (Apo-Laspau: *Lasioglossum pauxillum*, Apo-Colgig: *Colletes gigas*, Apo-Frivar: *Frieseomelitta varia*, Apo-Megrot: *Megachile rotundata*, Apo-Cercal: *Ceratina calcarata*, Apo-Seltum: *Seladonia tumulorum*, Apo-Anddor: *Andrena dorsata*, Apo-Holcal: Holcopasites calliopsidis, Apo-Exotri: *Exoneurella tridentata*, Apo-Ectcon: *Ectemnius continuus*, Apo-Mimdah: *Mimumesa dahlbomi*, Apo-Cteter: *Ctenoplectra terminalis*, Apo-Maceur: *Macropis europaea*, Apo-Andmin: *Andrena minutula*, Apo-Nysspi: *Nysson spinosus*, Apo-Hablab: *Habropoda laboriosa*, Apo-Bomter: *Bombus terrestris*, Apo-Megwil: *Megachile willughbiella*, Apo-Bomhae: *Bombus haemorrhoidalis*, Apo-Ampcom: *Ampulex compressa*). Multiple sequence alignments of other domains are in Supplementary Figure S2.

**Figure 6:**
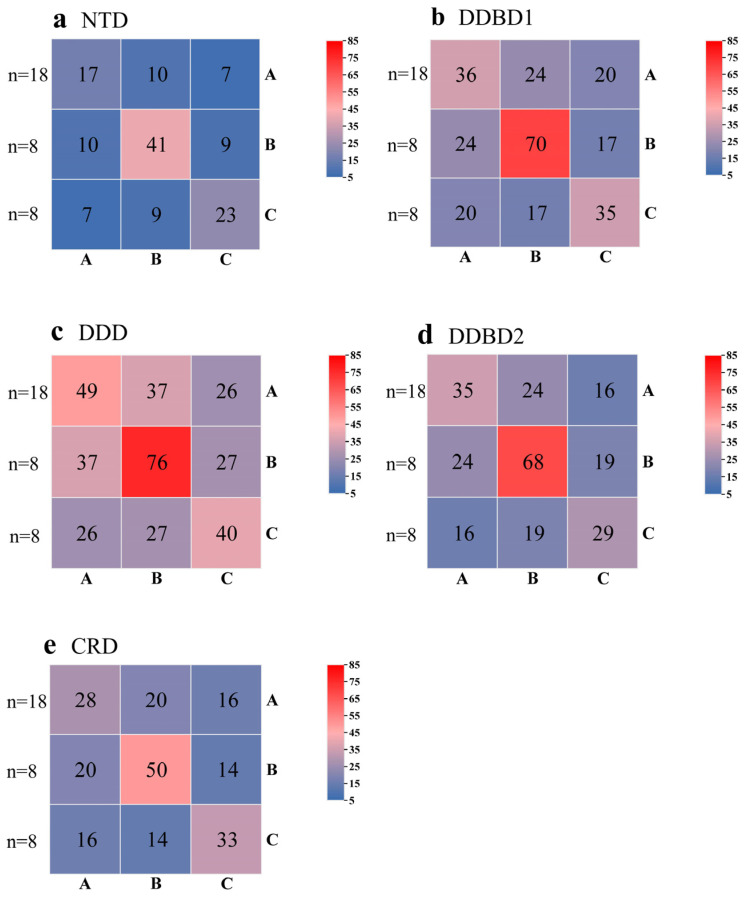
Sequence identities between A–C clades. The numbers in the heatmap are the percentage of the average values of the sequences’ identities of the two types of transposons in the corresponding row and column, and “*n*” represents the number of each type of transposon sequence (**a**–**e**). The average values of sequence identities were measured by pairwise comparison of sequences of NTD (**a**), DDBD1 (**b**), DDD (**c**), DDBD2 (**d**), and CRD (**e**) of complete *PB* transposases (≥500 aa).

*PB* transposases are known to contain five well-defined domains: the N-terminal domain (NTD), the Dimerization and DNA-binding domain 1 (DDBD1), the catalytic domain (also named DDE/DDD), DDBD2, and the C-terminal cysteine-rich domain (CRD) [45]. Sequence analysis showed that the catalytic domains are highly conserved (Figure 5) with high sequence identities within and between the clades (ranging from 40% to 76% and 26% to 37%, respectively) (Figure 6c), while other domains, including DDBD1, DDBD2, CRD, and NTD, are less conserved (Supplementary Figure S2A–D) and show low sequence identities within and between clades (Figure 6a,b,d,e), particularly the NTDs, which display very low sequence identities between clades (≤ 10%, Figure 6a) and are poorly conserved across clades (Figure 6a). The three key catalytic residues (DDD), crucial for catalyzing the transposition reaction [43,60,66], are highly conserved in the three clade transposases. The insertion motif, identified between the second and third key catalytic D residues [45,67], is conserved across the three clades, with slight variations observed for the C terminal of the insertion motifs of the clade C transposases (Figure 5). Additionally, CRD, required for *PB* activity and thought to be the driver of TIR binding [45], exhibits seven cysteines with regular spacing that are highly conserved in the CRD of the three clade transposases (Supplementary Figure S2D). Residues of P131-N152, E175-K190, and V207-D228 in DDBD1 are also conserved among the three clade transposases [68] (Supplementary Figure S2B). The N-terminal of DDBD2 contains a highly conserved tryptophan (W) known to play a central role in the transposase activity and DNA hairpin formation and resolution [43,44] (Supplementary Figure S2C). The DDBD and catalytic domain, with its insertion motif, are known to collaborate to synapse TIRs and direct the scissile phosphates to the active sites comprising D268, D346, and D447 [45].

### 3.5. Evolution Dynamics of piggyBac in the Apoidea Genomes

**Figure 7:**
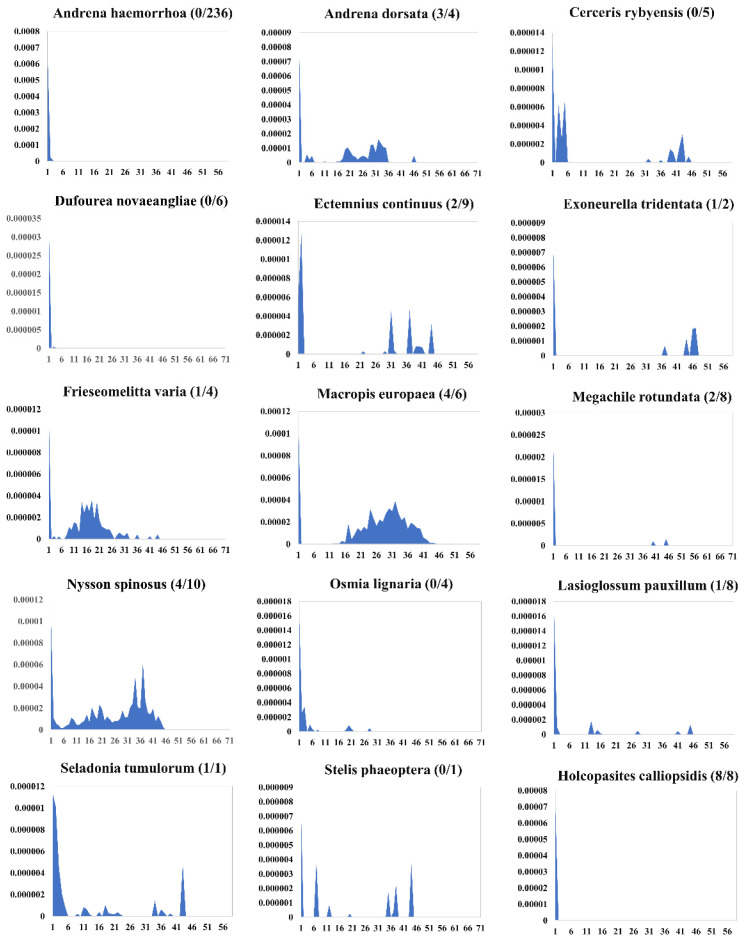
Evolutionary dynamics of *piggyBac* (*PB*) in Apoidea. The y-axis represents each *PB* transposon’s coverage (%) in the genome, and the x-axis indicates the K divergence estimate (%). In contrast, the numbers in brackets represent the intact copy number and total copy number of *PB* transposon in each genome. The figure only shows species with low K values. At the top of each chart are species names. More information is displayed in Figure S3.

To thoroughly investigate the evolutionary dynamics of the *PB* family in the genomes of Apoidea, we used RepeatMasker [63] to calculate the Kimura divergence of *PB* transposons in the *PB*-detected genomes. Divergent evolution dynamics of *PB* were observed in these species (Figure 7 and Supplementary Figure S3). Most copies of *PB* transposons in the genomes of fifteen species (*A. haemorrhoa*, *Cerceris rybyensis*, *Ectemnius continuus*, *Exoneurella tridentata*, *Holcopasites calliopsidis*, *Lasioglossum pauxillum*, *Macropis europaea*, *Seladonia tumulorum*, *A. dorsata*, *F. varia*, *Megachile rotundata*, *Osmia lignaria*, *Stelis phaeoptera*, *Dufourea novaeangliae,* and *Nysson spinosus*) represent K divergences of less than 2%, and seem to have invaded very recently. Furthermore, intact copies were detected in some species (*A. dorsata*, *E. continuus*, *E. tridentata*, *F. varia*, *M. europaea*, *M. rotundata*, *L. pauxillum*, *S. tumulorum*, *H. calliopsidis,* and *N. spinosus*) (Figure 7). These data indicated that *PB* transposons are young invaders in these bee genomes and may still have transposition activity.

On the other hand, PB copies in some species (*B. bifarius*, *Bombus hypnorum*, *Bombus haemorrhoidalis*, *C. calcarata, Ctenoplectra terminalis*, *E. robusta*, *Habropoda laboriosa*, and *Mimumesa dahlbomi*) show high K divergences (K > 15), suggesting that they may be old invaders in these lineages (Supplementary Figure S3) and may have lost their jumping activities. Additionally, multiple amplification waves were observed in *Bombus balteatus, Bombus huntii*, *B. bifarius*, *Bombus ignitus*, *C. calcarata*, *C. terminalis*, *Ampulex compressa*, *Andrena minutula*, *B. haemorrhoidalis*, *E. robusta*, *H. laboriosa*, *Nomada fabriciana*, *B. terrestris*, and *S. phaeoptera*, suggesting that these species might have experienced multiple invasions of PB transposon (Supplementary Figure S3).

## 4. Discussion

### 4.1. Distribution, Diversity and Copy Number of piggyBac in Apoidea

*PB* transposons are widely distributed in various vertebrates (Actinopterygii, Primate, and Rodentia) and invertebrates (Nematoda, Mollusca, Arthropods, Cnidaria, Stramenopiles, and Platyhelminthes) [17,35,36,69,70]. In our study, we found that *PB* transposons were detected in approximately one-third of bee species (34.65%, 44/127), and they were unevenly distributed across families and genera of bees. Notably, due to the high species diversity of *Bombus*, the number of *PB* transposons detected in *Bombus* was significantly higher than in other genera. Moreover, the transposons in *Bombus* were relatively conservative, with almost all *PB* transposons found in *Bombus* belonging to Clade B, suggesting a possible vertical propagation of *PB* transposons in *Bombus*. According to the available data, *PB* transposons have great diversity in arthropod species, and besides the *PB* family, *Tc1/mariner*, *Busters* and other transposons are found mostly in arthropods [71,72,73]. Here, we found that *PB* transposons also showed high diversity in Apoidea. Three distinct clades of *PB* transposases (A–C) were identified in Apoidea, with low sequence similarity among them. *Megachile*, *Bombus*, *Ceratina*, and *Andrena* all harbored two types of *PB* transposons. When determining the distribution, we considered *PB* transposons to be present in the genome when sequence similarity reached 80% and query coverage reached 40%. However, this approach may have led to the exclusion of short, truncated elements derived from *PB*, potentially underestimating the taxonomic distribution of *PB* transposons in Apoidea. In addition, due to the continuous update of genome sequencing data, new families are constantly appearing in the PB superfamily, meaning the number of *PB* transposon categories in Apoidea will be more than the currently identified, and their diversity may be higher than we predicted.

After invading the host genome, transposons undergo amplification, diversification, inactivation, and elimination. The presence of intact copies may indicate that transposons are in the amplification stage or have recently undergone amplification and may be active [74,75]. However, although *PB* transposons have invaded a large number of bee genomes, the total copy number is very low in most species, indicating that most *PB* transposons do not show significant amplification in bees. *Bombus*, which transitioned from solitary bees to social bees [12], has more copies than other genera, although the intact copies were relatively rare, suggesting that the transposon content may be somewhat related to the living environment. Most short copies or truncated copies of *PB*, due to lack of functional domains of transposases or TIRs, lost transposition activity. Other transposon families such as *ZB* and *SB*, which have been identified from animal genomes and belong to the *Tc1/mariner* superfamilies, exhibit high intact copy numbers (ranging from 10 to over 80 copies) in some genomes, with over 70% of species containing intact copies [76]. In contrast, intact copies of *PB* transposons are rare in Apoidea, with only 20 out of 44 species showing intact copies, and the copy numbers of intact copies are low in each detected species, with only *H. calliopsidis* having more than 5 intact copies in its genome, suggesting potential activity.

### 4.2. Structure Organization of piggyBac

The structure of most complete *PB* transposons in bees was found to be similar to the original sequence found in *T. ni*, consisting of five domains that have been previously defined [45]. TIRs play a crucial role in transposase recognition and target site cleavage [36,77,78]. Previous studies have shown that mutations in the first two pairs of bases in the TIRs can result in defects in the excision process [36,79]. However, our data showed that a large part of the identified *PB* transposons had CAC instead of CCC/GGG for their first three TIR bases. In seven bees (*A. haemorrhoa*, *B. balteatus*, *Bombus confusus*, *B. haemorrhoidalis*, *B. huntii*, *B. terrestris*, and *C. calcarata*), the TSDs of the *PB* transposons were not TTAA, which suggests possible mutations. Our data, along with previous studies, revealed that *PB* transposases may contain a highly divergent DDD domain, analogous to the widely distributed DDE domain found in many DNA transposases and retroviral integrases, which consists of two highly conserved aspartic acids and one glutamic acid [35]. The DDD domains of the three clades of transposases were highly conserved and are known to form an RNase H-like fold [43,60,66], with involvement in the nicking, hairpin resolution, and target joining of *PB* transposons [43,44]. Although the *PB* transposon in *M. dahlbomi* can encode protein of 596 aa with consistent 16 bp TIR at both ends, the second conserved residue in the catalytic domain of DDD mutated from “D” to “N”. Similarly, *PB* transposons in *A. compressa* encode a protein of 552 aa and have 14 bp TIR at both ends; the third conserved remnant in the catalytic domain of DDD mutated from “D” to “H”. These results suggest that *PB* transposons in these two species may have lost their transposable activity, which is supported by the late insertion age of transposons in *M. dahlbomi*, as determined by evolutionary dynamics analysis. In contrast, the DDBD1 and DDBD2 domains among the three types of *PB* transposons are not as conserved as DDD. DDBD1 and DDBD2 are special all-α-helix domains, knitting the protein together and interacting with TIR [45]. While the CRD of *PB* transposases are not conserved, they contain several highly conserved cysteines, with conserved distances between them. The CRD in the *PB* transposases do not match many other known C-rich domains [35]. NTD is the least conserved among all the domains, similar to other transposases, and may be involved in binding TIRs [35]. NTD residues show high variability, except for a more conserved methionine at the N-terminal and tryptophan at the C-terminal. Multiple sequence alignment indicates that Clade B transposons are more conserved than the other two clades.

### 4.3. Evolution Dynamics of piggyBac in Apoidea

Generally, differential evolutionary dynamics were observed for *PB* elements in Apoidea, with most of the excavated *PB* transposons representing old copies indicated by high K divergence. However, *PB* transposons in several species (such as *A. dorsata*, *E. continuus*, *E. tridentata*, *F. varia*, *M. europaea*, *M. rotundata*, *L. pauxillum*, *S. tumulorum*, *H. calliopsidis*, and *N. spinosus*) showed very low K divergences, indicating that these transposons are recent invaders and might still be active, contributing to ongoing genome renewal. In addition, multiple waves of *PB* amplifications were detected for some species, suggesting repeat invasions of *PB* transposons. The copy number of each transposon appears to be related to its insertion age and evolutionary mode. For instance, *PB* transposons in *B. hypnorum* and *B. bifarius* had relatively old insertion times, and the copies of *PB* transposons in these genomes tend to be degenerated due to the accumulation of mutations. In contrast, *PB* transposons in *H. calliopsidis* display very low K divergences, indicating young insertion ages, and more intact copies were detected.

## 5. Conclusions

Our study provides the first comprehensive information on the distribution of *PB* transposons in 44 species of Apoidea and has analyzed their activity, phylogeny, and structural characteristics. In general, *PB* transposons are widely distributed in Apoidea and can be divided into three classes. Despite low overall sequence similarity among the three clades, each class of transposons exhibits highly conserved DDD domains. Furthermore, we detected low copy numbers of *PB* transposons in most Apoidea genomes. Interestingly, we observed divergent evolution dynamics of *PB* transposons in the genomes of Apoidea, with intact copies being rare in these 44 species.

## Figures and Tables

**Table 1:** Full and intact copy numbers of *piggyBac* (*PB*) transposons in genomes of Apoidea.

Distribution ^a^	Ampulicidae	Andrenidae	Apidae	Colletidae	Crabronidae	Halictidae	Megachilidae	Melittidae
Full Copy Number	3	2–236	1–335	4	4–10	1–8	1–8	6
Average ^b^	3.00	61.25	30.00	4.00	7.00	5.00	4.75	6.00
Number of Species with Full Transposons	1	4	26	1	4	3	4	1
Intact Copy Number	1	0–3	0–8	1	0–4	0–1	0–3	4
Average ^C^	1.00	1.25	0.62	1.00	2.00	0.67	1.25	4.00
Number of Species with Intact Transposons	1	2	8	1	3	2	2	1

^a^ Distribution: eight families of Apoidea in which *PB* transposons were detected. ^b^ Average full copy (transposons flanked by detectable TSDs and TIRs) number of transposons in genomes of Apoidea. ^C^ Average intact copy (transposons flanked by detectable TSDs and TIRs and encoded ≥500 aa transposases) number of transposons in genomes of Apoidea.

**Table 2:** Structure organization of *piggyBac (PB*) transposons.

Distribution ^a^	Species Number	Length of Full Transposons (bp) ^b^	Number of Species with Full Transposons	Length of Intact Transposon (bp) ^C^	Number of Species with Intact Transposons	Transposase Length (aa)	TIR Length (bp)	TSD
Ampulicidae	1	2380	1	2380	1	552	14	TTAA
Andrenidae	4	2519–3518	4	2519–3518	2	83–681	5–23	TTAA
Apidae	26	368–3518	26	1670–3413	8	24–612	7–493	TTAA
Colletidae	1	2838	1	2838	1	589	16	TTAA
Crabronidae	4	2334–2712	4	2334–2712	3	449–601	13–18	TTAA
Halictidae	3	2326–2596	3	2326–2596	2	477–580	13–17	TTAA
Megachilidae	4	900–2868	4	2292–2868	2	206–581	15–20	TTAA
Melittidae	1	2646	1	2646	1	567	13	TTAA

^a^ Distribution: eight families of Apoidea in which *PB* transposons were detected. ^b^ Full transposon: transposons flanked by detectable TSDs (target site duplication) and TIRs. ^C^ Intact transposon: transposons flanked by detectable TSDs and TIRs and encoded ≥500 aa transposases.

## Data Availability

All data needed to evaluate the conclusions in this paper are present either in the main text or in the Supplementary Materials.

## Supplementary Materials

The following supporting information can be downloaded at: https://www.mdpi.com/article/10.3390/insects14040402/s1, Figure S1: Complete phylogenetic tree of *piggyBac* (*PB*) elements in Apoidea identified in this study together with 24 reference sequences. The scale bar of the phylogenetic tree represents 0.5. The sequences of IS1380 were used as the outgroup. Red, blue, and yellow circles, respectively, represented three types of *PB* transposons in Apoidea. (A–C). (Apo-Laspau: *Lasioglossum pauxillum*, Apo-Andful: *Andrena fulva*, Apo-Colgig: *Colletes gigas*, Apo-Cerryb: *Cerceris rybyensis*, Apo-Megrot: *Megachile rotundata*, Apo-Frivar: *Frieseomelitta varia*, Apo-Holcal: *Holcopasites calliopsidis*, Apo-Exotri: *Exoneurella tridentata*, Apo-Cercal: *Ceratina calcarata*, Apo-Seltum: *Seladonia tumulorum*, Apo-Anddor: *Andrena dorsata*, Apo-Ectcon: *Ectemnius continuus*, Apo-Mimdah: *Mimumesa dahlbomi*, Apo-Cteter: *Ctenoplectra terminalis*, Apo-Maceur: *Macropis europaea*, Apo-Andmin: *Andrena minutula*, Apo-Nysspi: *Nysson spinosus*, Apo-Hablab: *Habropoda laboriosa*, Apo-Bomter: *Bombus terrestris*, Apo-Bomvos: *Bombus vosnesenskii*, Apo-Megwil: *Megachile willughbiella*, Apo-Nomfab: *Nomada fabriciana*, Apo-Bomhae: *Bombus haemorrhoidalis*, Apo-Ampcom: *Ampulex compressa*, Apo-Dufnov: *Dufourea novaeangliae*, Apo-Stepha: *Stelis phaeoptera*, Apo-Eugdil: *Euglossa dilemma*, Apo-Bomtur: *Bombus turneri*, Apo-Bomhyp: *Bombus hypnorum*, Apo-Bomsor: *Bombus soroeensis*, Apo-Bombal: *Bombus balteatus*, Apo- Bomcon: *Bombus confusus*, Apo-Bomign: *Bombus ignitus*, Apo-Bombif: *Bombus bifarius*, Apo-Ceraus: *Ceratina australensis*). Figure S2: Multiple sequence alignment of different domains of three classes. Different conserved residues are colored differently: (A) Alignment of NTD of complete piggyBac (*PB*) transposases (≥500 aa). The black boxes are the more conservative areas; (B) Alignment of DDBD1 of complete *PB* transposases. The numbers above represent the position of the amino acid; (C) Alignment of DDBD2 of complete *PB* transposases; (D) Alignment of CRD of complete *PB* transposases. (Apo-Laspau: *Lasioglossum pauxillum*, Apo-Colgig: *Colletes gigas*, Apo-Frivar: *Frieseomelitta varia*, Apo-Megrot: *Megachile rotundata*, Apo-Cercal: *Ceratina calcarata*, Apo-Seltum: *Seladonia tumulorum*, Apo-Anddor: *Andrena dorsata*, Apo-Holcal: *Holcopasites calliopsidis*, Apo-Exotri: *Exoneurella tridentata*, Apo-Ectcon: *Ectemnius continuus*, Apo-Mimdah: *Mimumesa dahlbomi*, Apo-Cteter: *Ctenoplectra terminalis*, Apo-Maceur: *Macropis europaea*, Apo-Andmin: *Andrena minutula*, Apo-Nysspi: *Nysson spinosus*, Apo-Hablab: *Habropoda laboriosa*, Apo-Bomter: *Bombus terrestris*, Apo-Megwil: *Megachile willughbiella*, Apo-Bomhae: *Bombus haemorrhoidalis*, Apo-Ampcom: *Ampulex compressa*). Figure S3: Evolutionary dynamics of *piggyBac* (*PB*) in Apoidea. The y-axis represents the coverage (%) of each *PB* transposon in the genome, and the x-axis indicates the K divergence estimate (%), while the numbers in blankets represent the intact copy number and total copy number of *PB* transposon in each genome. The figure only shows species with high K values. At the top of each chart are species names. Table S1: Information about *piggyBac* (*PB*) elements in Apoidea.
