# Supplementary material for: Evolution of piggyBac Transposons in Apoidea
Source: Insects. 2023 Apr 21;14(4):402. doi: 10.3390/insects14040402 (PMC10140906; doi:10.3390/insects14040402)
Supplement: Supplementary file 1 [file insects-14-00402-s001.zip › insects-2306951-supplementary/Figure S1.pdf]

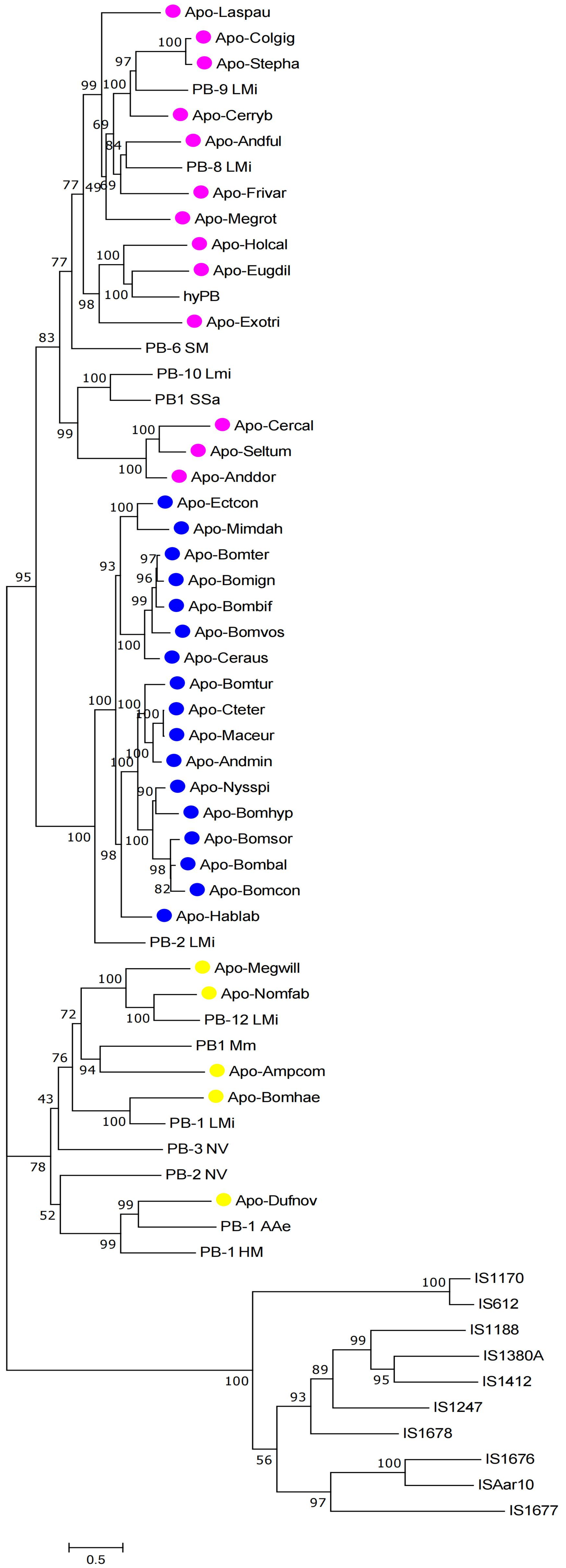

Figure S1. Complete phylogenetic tree of *piggyBac* (PB) elements in Apoidea identified in this study together with 24 reference sequences. The sequences of IS1380 were used as the outgroup. Red, blue and yellow circles, respectively, represented three types of PB transposons in Apoidea. (A, B, C). (Apo-Laspau: *Lasioglossum pauxillum*, Apo-Andful: *Andrena fulva*, Apo-Colgig: *Colletes gigas*, Apo-Cerryb: *Cerceris rybyensis*, Apo-Megrot: *Megachile rotundata*, Apo-Frivar: *Frieseomelitta varia*, Apo-Holcal: *Holcopasites calliopsidis*, Apo-Exotri: *Exoneurella tridentata*, Apo-Cercal: *Ceratina calcarata*, Apo-Seltum: *Seladonia tumulorum*, Apo-Anddor: *Andrena dorsata*, Apo-Ectcon: *Ectemnius continuus*, Apo-Mimdah: *Mimumesa dahlbomi*, Apo-Cteter: *Ctenoplectra terminalis*, Apo-Maceur: *Macropis europaea*, Apo-Andmin: *Andrena minutula*, Apo-Nysspi: *Nysson spinosus*, Apo-Hablab: *Habropoda laboriosa*, Apo-Bomter: *Bombus terrestris*, Apo-Bomvos: *Bombus vosnesenskii*, Apo-Megwil: *Megachile willughbiella*, Apo-Nomfab: *Nomada fabriciana*, Apo-Bomhae: *Bombus haemorrhoidalis*, Apo-Ampcom: *Ampulex compressa*, Apo-Dufnov: *Dufourea novaeangliae*, Apo-Stepha: *Stelis phaeoptera*, Apo-Eugdil: *Euglossa dilemma*, Apo-Bomtur: *Bombus turneri*, Apo-Bomhyp: *Bombus hypnorum*, Apo-Bomsor: *Bombus soroeensis*, Apo-Bombal: *Bombus balteatus*, Apo- Bomcon: *Bombus confusus*, Apo-Bomign: *Bombus ignitus*, Apo-Bombif: *Bombus bifarius*, Apo-Ceraus: *Ceratina australensis*)
