# Supplementary material for: Evolution of piggyBac Transposons in Apoidea
Source: Insects. 2023 Apr 21;14(4):402. doi: 10.3390/insects14040402 (PMC10140906; doi:10.3390/insects14040402)
Supplement: Supplementary file 1 [file insects-14-00402-s001.zip › insects-2306951-supplementary/Figure S2.pdf]

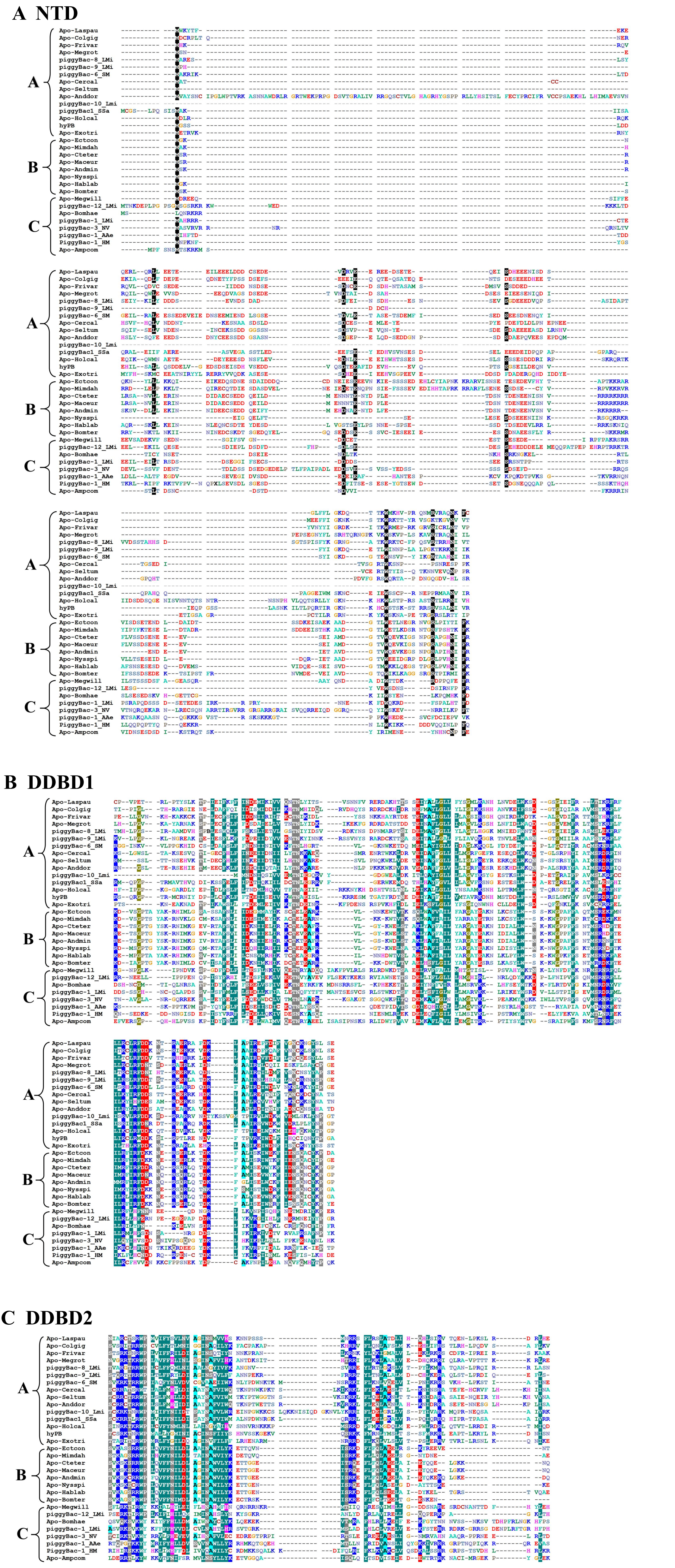

Figure S2. Multiple sequence alignment of different domains of three classes. Different conserved residues are colored differently. (A) Alignment of NTD of complete *piggyBac* (PB) transposases (≥500aa). The black boxes are the more conservative areas (B) Alignment of DDBD1 of complete PB transposases. (C) Alignment of DDBD2 of complete PB transposases. (D) Alignment of CRD of complete PB transposases. (Apo-Laspau: *Lasioglossum pauxillum*, Apo-Colgig: *Colletes gigas*, Apo-Frivar: *Frieseomelitta varia*, Apo-Megrot: *Megachile rotundata*, Apo-Cercal: *Ceratina calcarata*, Apo-Seltum: *Seladonia tumulorum*, Apo-Anddor: *Andrena dorsata*, Apo-Holcal: *Holcopasites calliopsidis*, Apo-Exotri: *Exoneurella tridentata*, Apo-Ectcon: *Ectemnius continuus*, Apo-Mimdah: *Mimumesa dahlbomi*, Apo-Cteter: *Ctenoplectra terminalis*, Apo-Maceur: *Macropis europaea*, Apo-Andmin: *Andrena minutula*, Apo-Nysspi: *Nysson spinosus*, Apo-Hablab: *Habropoda laboriosa*, Apo-Bomter: *Bombus terrestris*, Apo-Megwil: *Megachile willughbiella*, Apo-Bomhae: *Bombus haemorrhoidalis*, Apo-Ampcom: *Ampulex compressa*)
