# Supplementary material for: Evolution of piggyBac Transposons in Apoidea
Source: Insects. 2023 Apr 21;14(4):402. doi: 10.3390/insects14040402 (PMC10140906; doi:10.3390/insects14040402)
Supplement: Supplementary file 1 [file insects-14-00402-s001.zip › insects-2306951-supplementary/Figure S3.pdf]

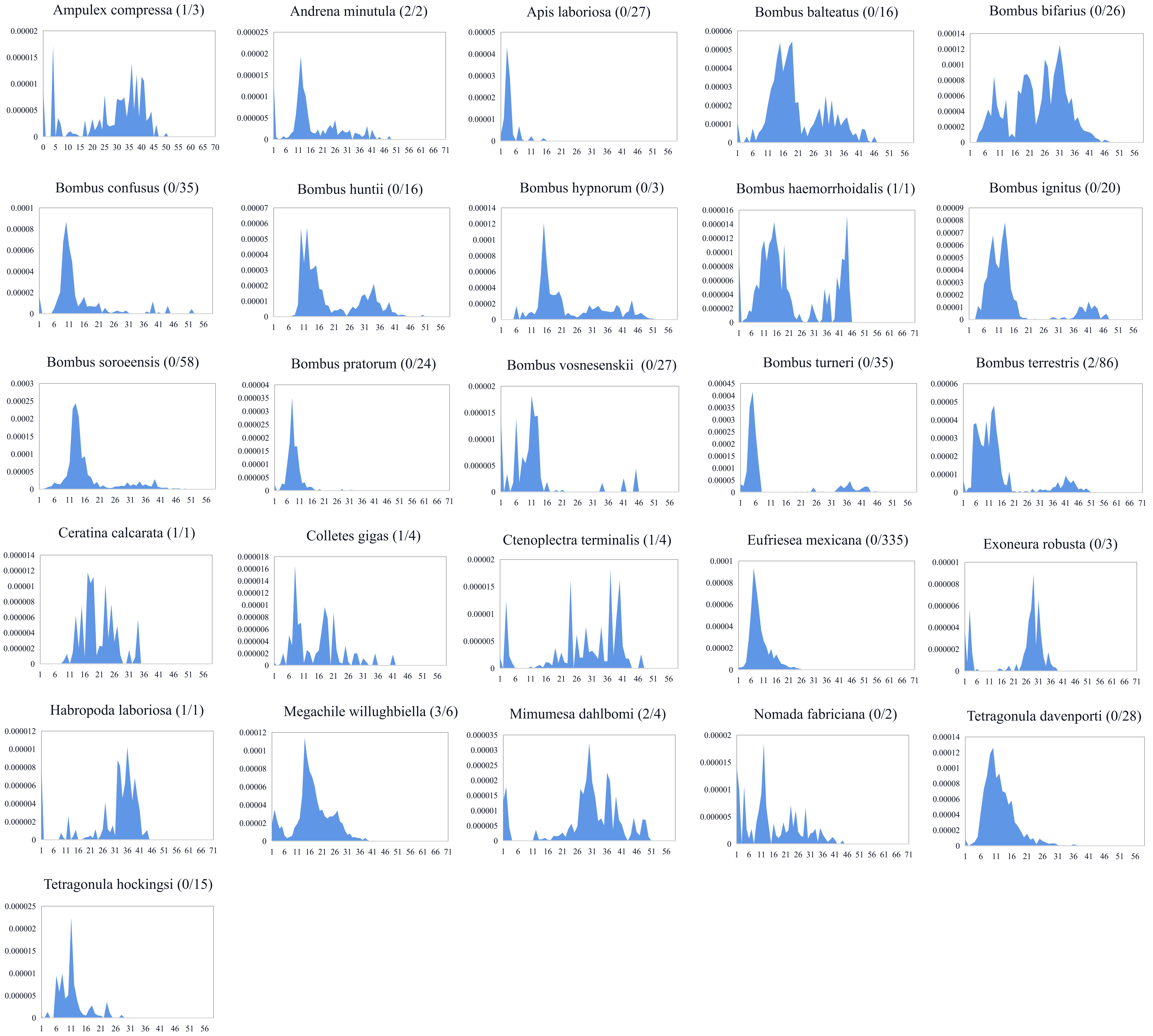

Figure S3. Evolutionary dynamics of *piggyBac* (PB) in Apoidea. The y-axis represents the coverage (%) of each PB transposon in the genome, and the x-axis indicates the K divergence estimate (%), while the numbers in blankets represent the intact copy number and total copy number of PB transposon in each genome. The figure only shows species with high K values. At the top of each chart are species names.
