# Supplementary material for: Evolution of piggyBac Transposons in Apoidea
Source: Insects. 2023 Apr 21;14(4):402. doi: 10.3390/insects14040402 (PMC10140906; doi:10.3390/insects14040402)
Supplement: Supplementary file 1 [file insects-14-00402-s001.zip › insects-2306951-supplementary/Table S1.pdf]

Superfamily	Family	Species	Length (bp)	ORF (aa)	Copy number (>80% identity and > 40% coverage)	Copy number of intact Tns (≥500)	Size (bp) Left/Right	TIR (L/R) Identity %	TIR end (5 bp)	TSD
Apoidea	Ampulicidae	*Ampulex compressa*	2380	552	3	1	14/14	85.71	CCGTT/TCGTT	TTAA
Apoidea	Andrenidae	*Andrena dorsata*	3518	681	4	3	5/5	100	CCCTC/CCCTC	TTAA
Apoidea	Andrenidae	*Andrena fulva*	2581	457	3	0	17/17	100	CCCGG/CCCGG	TTAA
Apoidea	Andrenidae	*Andrena haemorrhoa*	777	83	236	0	23/23	100	GGTAA/GGTAA	TAA
Apoidea	Andrenidae	*Andrena minutula*	2519	560	2	2	13/13	100	CACTA/CACTA	TTAA
Apoidea	Apidae	*Apis laboriosa*	471	32	27	0	14/14	92.86	CCCTT/TCCTT	TTAA
Apoidea	Apidae	*Bombus bifarius*	3518	204	26	0	201/191	87.56	CACTA/CACTA	TTAA
Apoidea	Apidae	*Bombus hypnorum*	2767	243	3	0	16/16	100	CACTA/CACTA	TTAA
Apoidea	Apidae	*Bombus soroeensis*	2750	182	58	0	14/14	92.86	CACTA/TACTA	TTAA
Apoidea	Apidae	*Bombus vosnesenskii*	1670	357	27	0	15/15	100	CACTA/CACTA	TTAA
Apoidea	Apidae	*Bombus balteatus*	2468	184	16	0	16/16	93.75	CACTA/CACCA	TAA
Apoidea	Apidae	*Bombus confusus*	1942	142	35	0	7/7	85.71	CACTA/CCCTA	TT-A
Apoidea	Apidae	*Bombus haemorrhoidalis*	2288	555	1	1	19/19	73.68	CCTTA/CCTTT	TAT
Apoidea	Apidae	*Bombus huntii*	1869	96	16	0	13/13	92.31	CACTA/CACTA	TAA
Apoidea	Apidae	*Bombus ignitus*	2909	215	20	0	16/16	100	CACTA/CACTA	TTAA
Apoidea	Apidae	*Bombus pratorum*	610	86	24	0	13/13	92.31	CACGT/CACGT	TTAA
Apoidea	Apidae	*Bombus terrestris*	2713	547	86	2	200/200	95.5	CGGGA/CGGGA	TT-A
Apoidea	Apidae	*Bombus turneri*	2478	340	35	0	11/11	100	CACTA/CACTA	TTAA
Apoidea	Apidae	*Ceratina australensis*	2718	310	2	0	15/15	93.33	CACTA/CACTA	TTAA
Apoidea	Apidae	*Ceratina calcarata*	3413	566	1	1	14/12	78.57	CGAGT/CGAGT	T

Apoidea	Apidae	*Ctenoplectra terminalis*	2645	567	4	1	13/13	100	CACTA/CACTA	TTAA
Apoidea	Apidae	*Eufriesea mexicana*	450	93	335	0	12/12	91.67	CCCAT/CCCAC	TTAA
Apoidea	Apidae	*Euglossa dilemma*	2435	220	1	0	14/14	85.71	CCTAG/CCCAG	TTAA
Apoidea	Apidae	*Exoneura robusta*	1289	24	3	0	493/489	96.96	CGCCG/CGCCG	TTAA
Apoidea	Apidae	*Exoneurella tridentata*	2589	587	2	1	17/17	88.24	CCTTC/CTCTT	TTAA
Apoidea	Apidae	*Frieseomelitta varia*	3064	578	4	1	15/15	73.33	TTTTG/GTTTG	TTAA
Apoidea	Apidae	*Holcopasites calliopsidis*	2569	612	8	8	14/14	100	CCCTA/CCCTA	TTAA
Apoidea	Apidae	*Habropoda laboriosa*	2422	583	1	1	13/13	100	CACTA/CACTA	TTAA
Apoidea	Apidae	*Nomada fabriciana*	2477	474	2	0	18/18	100	CACTT/CACTT	TTAA
Apoidea	Apidae	*Tetragonula davenporti*	1374	81	28	0	14/14	85.71	CCCTT/CTCTT	TTAA
Apoidea	Apidae	*Tetragonula hockingsi*	368	36	15	0	12/12	100	CCCTT/CCCTT	TTAA
Apoidea	Colletidae	*Colletes gigas*	2838	589	4	1	16/16	100	CCTTC/CCTTC	TTAA
Apoidea	Crabronidae	*Cerceris rybyensis*	2334	449	5	0	18/18	94.44	CCTTT/CCTTT	TTAA
Apoidea	Crabronidae	*Ectemnius continuus*	2519	601	9	2	15/15	100	CACTA/CACTA	TTAA
Apoidea	Crabronidae	*Mimumesa dahlbomi*	2667	596	4	2	16/16	100	CACTA/CACTA	TTAA
Apoidea	Crabronidae	*Nysson spinosus*	2712	535	10	4	13/13	100	CACTA/CACTA	TTAA
Apoidea	Halictidae	*Dufourea novaeangliae*	2596	477	6	0	15/15	93.33	CCCTT/CCCTT	TTAA
Apoidea	Halictidae	*Lasioglossum pauxillum*	2331	580	8	1	17/17	100	CCCGC/CCCGC	TTAA
Apoidea	Halictidae	*Seladonia tumulorum*	2326	567	1	1	13/13	92.31	CCCTC/CCCTC	TTAA
Apoidea	Megachilidae	*Megachile willughbiella*	2868	569	6	3	15/15	100	CCCAT/CCCAT	TTAA
Apoidea	Megachilidae	*Megachile rotundata*	2374	581	8	2	17/17	94.12	CCCTT/CCCTT	TTAA
Apoidea	Megachilidae	*Osmia lignaria*	900	62	4	0	15/15	93.33	CCCTC/CCCTC	TTAA
Apoidea	Megachilidae	*Stelis phaeoptera*	2292	206	1	0	20/20	100	CCTTC/CCTTC	TTAA
Apoidea	Melittidae	*Macropis europaea*	2646	567	6	4	13/13	100	CACTA/CACTA	TTAA

Table S1. Information about *piggyBac* (PB) elements in Apoidea.
